# Qua Iboe by motorcycle and launch: brokering public health coverage in 1960s Southeastern Nigeria

**DOI:** 10.1080/13507486.2021.1958762

**Published:** 2022-01-26

**Authors:** John Manton

**Affiliations:** Centre for History in Public Health, London School of Hygiene and Tropical Medicine, London, UK

**Keywords:** Public health, leprosy control, Nigeria, decolonization, health workers

## Abstract

Long an expatriate-run concern, leprosy control was subsumed as a key component of rural public health in the years following Nigerian Independence in 1960 by the enlisting of a cadre of African inspectors, deployed across an existing institutional landscape by a newly Nigerianized medical bureaucracy. The performative norms of leprosy control, once thoroughly colonial and suffused with the ripe vocabulary of a long-entrenched missionary diaspora, were renovated at the heart of a new concern with rural public health more broadly, as the needs, expectations and hierarchies encoded in relations between patient, court, bureaucrat and medical worker shifted and settled in accordance with new political horizons. For health workers, issues of patient and worker mobility, drug delivery, patient and community expectation, and their own physical and financial security were dramatized in a series of commentaries, complaints and reports denoting deeply felt anxieties over the viability of careers in the service of Nigerian health. This article outlines struggles surrounding leprosy control and rural public health work in the Qua Iboe Mission catchment, administered by the newly created Ikot Ekpene Medical Field Unit. It documents a shift in medical work from European missionary to national and technocratic, the foregrounding of concerns with African (worker and patient) welfare and mobility, and the emergence of novel post-colonial forms of public health advocacy and politics along the highways and byways of Ibibio- and Annang-speaking areas of southeastern Nigeria in the 1960s.

## Introduction

This article is concerned with the gradual and partial transfer of control of a prominent public health intervention in southeastern Nigeria from British colonial to African hands in the period immediately following Nigerian Independence in 1960, seen through the experiences of a cohort of African public health workers. It describes a shift in the framing of medical and rural public health work – specifically, the control of leprosy – from European missionary to national and technocratic character, the foregrounding of concerns with African (worker and patient) welfare and mobility, and the emergence of novel post-colonial forms of public health advocacy and politics along the highways and byways of Ibibio- and Annang-speaking areas of southeastern Nigeria in the 1960s. It documents a Nigerian instance of the managed transition to national delivery of health care programmes conceived and developed in the colonial era, examining the role of religion (specifically missionary health care), the challenges of financing as evidenced by elements of career building and labour precarity, and the problems encountered in extending health services equitably across a territory with an uneven distribution of population, ill health, medical establishments and transport infrastructure.

The Annang and Ibibio regions of colonial and early postcolonial Calabar Province,^1^ coterminous with the Kwa Ibo River Basin in present-day Akwa Ibom State, remote from colonial trading and population centres and crisscrossed with waterways, had been evangelized by the Qua Iboe Mission (QIM) since 1887.^2^ The Mission, an interdenominational Protestant enterprise based in Belfast with a strongly Presbyterian ethic, was established in the late nineteenth century around the missionary work of Samuel Bill in Ibeno, a coastal area near the mouth of the Kwa Ibo River. It played a prominent role in unfolding patterns of accommodation between Annang and Ibibio communities and the armed and mercantile incursions of the British colonial state.^3^

While the dislocations of colonial rule in southeastern Nigeria took place over a relatively short period of time, European involvement in slave and commodity trading in the Kwa Ibo River Basin and neighbouring coastal and riverine areas from the Cross River to the Niger Delta dated back to the sixteenth century, when the presence of British and Portuguese traders began to exert an external stimulus on trading, kinship and military–political networks in the region. Across the region, political, trading, farming and cultural–linguistic groups alternately took advantage of the various opportunities created by the export-led demand for slave labour, and sought to shelter from the political turmoil that resulted.^4^ Direct imperial military incursions across southeastern Nigeria in the first decades of the twentieth century sought to increase British influence in the conduct and regulation of agricultural production, labour control, and trade in the area, leading to a rapid recalibration of political, cultural/religious, and economic strategies open to Nigerians.^5^

In common with other Christian missionary groups capitalizing on colonial disruption and cultural and economic crisis in early twentieth-century southeastern Nigeria, the Qua Iboe Mission rapidly became involved in educational and medical provision. The Mission founded a hospital at Etinan, on the Eastern banks of the Kwa Ibo River, in 1927. Noticing cases of leprosy infection among patients treated at the hospital, the Mission leased land by the river to the north of Etinan, at Ekpene Obom, and began ‘combing out cases’, isolating and treating leprosy patients there from 1932 or 1933.^6^ Administered by a relatively small and underfunded mission, and close to prominent leprosy institutions at Itu and Uzuakoli, the leprosy work at Ekpene Obom struggled to take root until the early 1950s, but was then able to capitalize on closer engagement with local government, a grant from the Nigeria Leprosy Service, and an increase in the availability of trained leprosy workers.^7^ This put the Mission and its work at the heart of the shifting politics of leprosy control and its administration in the early years of Nigerian Independence.

## Materials and methods

This discussion, of the Africanization of the leprosy control bureaucracy in the Qua Iboe Mission sphere of influence, builds on material assembled in Nigeria in 2000, 2006, 2011–12 and 2015. It draws on files of administrative correspondence assembled at Qua Iboe Church Leprosy Hospital, Ekpene Obom, Akwa Ibom State (hereafter referred to as the Ekpene Obom files) which were examined on site in January 2006, archival material at the Nigerian National Archives, Ibadan and Enugu, and interviews conducted in Arochukwu, Abia State in 2015. Further investigations were carried out in 2019 at World Health Organization (WHO) Headquarters in Geneva, and in 2020 at the WHO African Regional Offices in Brazzaville. Material gathered in 2000 at the Medical Missionaries of Mary Convent, Ogoja (Ogoja Convent files) in connection with leprosy control in Ogoja Province, has also been of relevance in this discussion.

Key personnel involved in rural leprosy control outreach work in late 1950s and 1960s western Calabar Province were identified through analysis of the Ekpene Obom files, which were grouped loosely according to administrative function or local administrative/county council area. The interactions of these key personnel, both with one another and with missionary or government personnel, were coded in relation to themes of mobility, pay and appointments, expenses and allowances, and practical and legal impediments to fulfilment of their duties. Among the impediments noted were patient resistance to aspects of treatment or the organization of leprosy control, failures in funding and prompt payment, access to training and support, shortage of materials and apparatus for mobile disease control work and treatment, and legal disputes about aspects of treatment and control.

Analysis of materials consulted in branches of the Nigerian National Archives, and at WHO HQ and AFRO Regional Archives, was also undertaken. Coverage of the then Calabar Province through the most relevant period, from 1956 to 1967, is poor in all of these archives. Coverage of the colonial period is stronger than the postcolonial in the Nigerian Archives as a consequence of historic collection policies and practices, and where files remain open across the Independence period, there is little added after 1960.^8^ World Health Organization archival materials – engaging at a national level with newly independent African states – can only dimly illuminate the quotidian work of the new rural public health corps emerging from the context of colonial leprosy control in southeastern Nigeria.

## Framing labour in public health at the end of empire: between missionary medicine, colonial leprosy control and independent Nigerian public health

The literature on late colonial and postcolonial public health and welfare is growing rapidly. Histories of many of the functional components of a *de facto* approach to planning, financing and systematizing the provision of health services have begun to emerge in relation to specific services, professional cohorts and territories. It is now possible to sketch in outline the ways in which many of these components integrate to facilitate the consolidation of health care provision, relating territorial and national developments to the shifting politics of international public health in the period of rapid decolonization during the 1950s and 1960s.

Christian missionaries were central to the provision and extension of rural health services in much of colonial Africa and Asia – including the Nigerian case examined in this article – and missionary reflections on and adaptations to decolonization found a broader audience through the World Council of Churches (founded in Geneva in 1948 – the Catholic Church is not a member but has established joint working groups), and its Christian Medical Commission (founded in 1968), both of which liaised closely with the World Health Organization during the formulation of primary health care recommendations in the 1970s.^9^ Meanwhile, concern with health services financing and the strengthening of health systems was gathering within the World Health Organization as the membership grew with the admission of newly independent countries. It consolidated around a number of prominent programmes and interventions, developing alongside its own scholarship and conceptual apparatus based around comparison and systems analysis.^10^

Other internationally driven interventions which strengthened health care capacity at the level of the locality included prominent disease control programmes relating to malaria, yaws and smallpox. Bhattacharya and Campani complicate the historiographical politics of the Smallpox Eradication Programme, announced at the World Health Assembly in 1958. The authors describe the smoothing effect of dominant global (WHO and CDC) narratives of eradication, and contest this with a depiction of the micropolitics of disease control planning, failures and diplomacy in Latin America in the 1960s, concluding that the design and implementation of the ostensibly global eradication programme relied on negotiations driven at the WHO Regional Office level and in member state health bureaucracies, and was continually refined amid negotiated solidarities between health workers and diplomats.^11^

While many visions and accounts of postcolonial health planning locate the drivers of change at the national and international level, a substantial proportion of the labour required to parse, shape, implement and operationalize the functioning of health services has happened at the interface with patients and their communities.^12^ The parsimony and close attention to cost control which characterized the British colonial state in Nigeria, and continued to constrain health and welfare policy in the years immediately following Independence, meant that granular local contestation of the scope, siting and cost of services very strongly determined public health and health planning outcomes in different parts of Nigeria.^13^ Colonial health care provision was largely confined to tertiary hospital services in larger towns and cities, small, poorly staffed town hospitals, occasional dispensary services, and vector and infectious disease control programmes, often on a pilot basis. The often limited and compromised reach of campaigns conceived nationally or regionally, and the local clamour for specific medical services, together made up a complex and opaque policy terrain in health.

Evidence of this can be seen in British colonial engagements with WHO-led disease control. In the case of late colonial Eastern Nigeria, the most prominent transnational disease control effort was the Global Yaws Eradication Programme, devised and funded by WHO and UNICEF, and begun in Nigeria in 1952. The United Kingdom, in common with other European colonial powers, had kept the League of Nations Health Organization at arm’s length on questions of colonial health, but it began to engage tentatively with the WHO in the early 1950s, harnessing international funding and technical assistance to complement its own poorly funded colonial development programmes in health.^14^ Nigerian Medical Field Units had conducted disease prevalence surveys from 1947, identifying a high prevalence of yaws in rural areas, and a plan of operations was devised by the Government of Nigeria, together with WHO and UNICEF, taking account of local conditions, staffing and anticipated implementation difficulties. Significantly, implementing staff was drawn from prior sleeping sickness and medical survey campaigns, with leprosy inspectors drawn in to identify hitherto untreated cases of leprosy.^15^ For the Eastern Nigeria Regional government in Enugu, leprosy and yaws control were the only projects accorded the highest priority for WHO support in its comments on the 1956 UN Expanded Programme of Technical Assistance for Nigeria.^16^

Yaws control greatly expanded a previously *ad hoc* disease control and rural public health bureaucracy, giving it institutional purchase through large and complex mobile field units, and enabling the consolidation of vaccination, epidemiological and health education functions alongside field unit operations. Its complementarity with leprosy control work, materialized in skin inspection and rural health emphases, gave rise to a fusion of colonial government funded public health and missionary medicine, which played out in the working lives of a cohort of Nigerian medical attendants, auxiliaries, bureaucrats and inspectors.^17^ This process echoed experiences across colonial Africa, where the work of translation and interpretation carried out by medical auxiliaries greatly influenced the character and reach of biomedicine, infusing it with the stamp of the tools and resources with which it was carried out.^18^

The signal contribution of leprosy control, in this changing and internationalizing terrain of rural public health after 1945, was to mobilize the rhetorical energies and transnational humanitarian networks of Christian missionary organizations, and to provide a template for the engagement of both colonial and international bodies with a medical programme which resonated with European and African Christians alike. In response to the high prevalence of leprosy in Eastern Nigeria – estimated at up to 10% of the population in some rural areas by the 1940s and early 1950s – the colonial government committed almost as much of its budget to developing the infrastructure and bureaucracy of leprosy control as to all other medical services together. This bureaucratization built on missionary coordination, networks, institutions and staffing and training programmes, which formed the core of the government’s Nigeria Leprosy Service founded in 1945.^19^ This massive and increasingly coordinated response to the prevalence and perceived threat of leprosy fused existing transnational networks with advances in chemotherapy, and capital-intensive and highly regulated institutions for treatment and experimentation, sitting at the heart of a consolidated case-finding programme, which expanded throughout the late colonial and early Independence era, and relied on the advocacy, mobility and sensitivity of a cohort of trained African health workers.^20^

The success of missionary enterprises, whether viewed from the perspective of evangelism or of social provision, depended on insinuating these enterprises within the livelihood, welfare and therapeutic strategies of communities and individuals enduring the disruptive effects of recent colonial incursions into local economies and polities. These processes did not unfold according to the will of the colonizer or missionary alone, and depended from the outset on an emergent and adaptative colonial politics which was often fractious in the extreme. As David Pratten documents in relation to the painful and strenuous cultural and political adaptation to colonial disruption in the Annang areas of Calabar Province, the level of constraint applied under colonial governance could not erase this fundamental dimension of engagement.^21^

Missionary organizations constituted themselves across national boundaries, though they operated differently within each polity. The identification of missionaries with broader programmes of health strengthening draws on their self-conscious identification with bureaucratic stewardship in health, and a powerful current of cultural work linking therapeutic outcomes with redemptive arcs, and medical work with notions of sacrifice and spiritual love.^22^ In this, we can see clear parallels with frontier missionary work in Canada, described in the article by Vandenberg and Gallagher-Cohoon in this volume, where nation-building, cultural labour in assimilation, and welfare provision beyond state policy reach was also carried out by Christian missionary agents.

Catholic medical missionaries in British colonial territories benefitted from access to training and professional development in a system of hospitals which had substantial state support in post-Independence Ireland; the institutional analogies between imperial hospitals run by Catholic missionaries, and those in which they worked in Ireland, were stronger than among most Protestant missionaries, which had pragmatic on-the-ground implications for the interaction between missionary and government medical and public health work in different parts of southeastern Nigeria.^23^ There is not just one story of the unfolding of public health provision even across this relatively small geographical area through the middle and latter parts of the twentieth century. Though missionaries were in retreat by 1960 (Ogbu Kalu writes of the ‘dishevelled’ nature of missionary and colonial responses to decolonization),^24^ in relation to missionary work, this is most clearly legible in evangelism, where expatriate control was most easily supplanted by indigenous ministry. Capital-intensive work in medical and health sectors continued to bear the stamp of missionary control, in funds and personnel, and in the interests of maintaining an underlying continuity in provision.

## Leprosy control in Eastern Nigeria, integration with outpatient public health and the changing profile of labour

Since the mid-1930s, the principle of local support for leprosy control had been established for southeastern Nigeria, to be administered at the level of the native administration and clan court. This replaced a centralized model that had begun to build around the success of the Church of Scotland leprosy settlement at Itu in attracting the attention of British donors and colonial administrators in Nigeria. As new settlements were opened by Christian missionaries at Oji River (Anglican), Uzuakoli (Methodist), Ekpene Obom (Qua Iboe Mission) and Ogoja (Catholic), case finding and support for the segregation of patients began to be organized on a clan-based model, as recommended by Ernest Muir, a British Empire Leprosy Relief Association (BELRA) specialist who visited Nigeria in 1936.^25^ Over the subsequent two decades, the colonial state and missionary organizations invested heavily in the control of leprosy, which became perhaps the most significant investment in health and medicine in the Eastern Region of Nigeria under colonial rule.^26^

The growth of the government’s Nigeria Leprosy Service, established in 1945 with its headquarters in the southeast, greatly enlarged the scope of leprosy control, progressively bringing it into closer relation with broader medical and public health work (though stopping short of effective integration during the colonial period), and increasing opportunities for a civil service career pathway in leprosy control. The administrative headquarters of the Nigeria Leprosy Service were at Oji River, near Enugu, while the Leprosy Research Unit, operated by the Service and BELRA, was stationed at Uzuakoli, which occupied a central location in southeastern Nigeria, and had radial status in the network of large leprosy centres. There was a further administrative station at Ossiomo, Benin Province, which covered the southwest. The founding of a Leprosy Inspectors Training School at Oji River rationalized training and the structure of promotions within the developing bureaucracy of leprosy control, with leprosy attendant, inspector and control officer grades aligned to civil service salary scales.

The case finding apparatus in leprosy control was greatly strengthened by investment in an apparatus for yaws survey and control, supported by WHO and UNICEF from 1954, and slowly extending across southeastern Nigeria in the subsequent decade.^27^ The level of investment and high profile of the yaws eradication campaign helped build the groundwork for an effective separate, non-missionary basis for leprosy control within the apparatus for delivering rural public health. The campaign committed the Regional Government to train and provide key personnel, and interface with medical officers of health and Nigeria-based research institutes.^28^ It also uncovered a substantial number of new leprosy cases, including 1760 new cases in Nsukka Division, north of Enugu, among an examined population of 117,315.^29^ This focused attention further on the need to develop an inspectorate largely independent of the central leprosy settlements, which were in themselves under pressure to redefine as outpatient and referral centres through this period.

From the mid-1950s, the widespread adoption of dapsone for the treatment of leprosy led to a focus on outpatient treatment, and a diminution of the importance of residential treatment (and thus close control over patient movement) at the central settlement. This institutional and spatial dispersion introduced new organizational pressures for leprosy control. The central settlements, once the organizational core of all local work in leprosy control, increasingly assumed a referral status in the broader apparatus of leprosy control as a component of rural public health. A significant cohort of leprosy inspectors had been recruited across southeastern Nigeria and trained at the Leprosy Inspectors Training School at the Oji River headquarters of the Nigeria Leprosy Service. From 1956, this school had been rebranded as a Rural Health Training School, with the intention that leprosy attendants and inspectors would be absorbed into a newly transformed Rural Health Service, evolving out of the dispensary system which ran in parallel to leprosy control, and to a network of government and missionary hospitals.^30^ This echoed intuitions which underlay the rationale for Muir’s 1936 proposals for leprosy control, that leprosy could be a key disease in spreading notions of proper diet and sanitation among the rural population, as well as in providing a location in which treatment for a wide range of ancillary conditions could be administered.^31^

Further organizational strain in the bureaucratic apparatus of leprosy control came in the guise of the Yaws Eradication Programme, which diluted command structures and case finding in leprosy and rural health through the 1950s and into the 1960s, taking much of the direction of leprosy control out of the hands of the missionaries who had shaped the enterprise over the previous quarter century. Hand in hand with the shifting geography of increasingly outpatient leprosy control went a newly secular chain of command and accountability to regional and local government, which redefined the nature of inspection labour for a cohort of mobile workers across leprosy prevalent areas of southeastern Nigeria.

## Labour in leprosy control, brokering in rural health and extending healthcare coverage in the Kwa Ibo River Basin

While there were opportunities for women in missionary medicine and rural public health care, the ranks of leprosy attendants, inspectors and control officers seem to have been male almost without exception. These men travelled across the counties and provinces of southeastern Nigeria carrying out leprosy and yaws surveys, bargaining with local chiefs and county administrations, and dealing with patient referrals, queries and complaints. In Annang and Ibibio areas administered by the QIM Leprosy Settlement, men such as Isaac O. Onoh, A.O. Ubbor, O.A. Mbah, O.T. Umoh, Fred Akpan, Emmanuel Enang, S.I. Adim and A.K. Udochu transacted leprosy control throughout the catchment.

For these men, the pathway to and through a career working in leprosy control changed in relation to bureaucratic and professional shifts in government service and increasing oversight on the conduct of public health work. In the early years of the expansion of leprosy control in southeastern Nigeria, the central provincial leprosy colony or settlement commanded most of the attention and investment from missionaries and their donor constituencies, and from the colonial state. Senior staff tended to be expatriate, and avenues for training were vested in the individual settlement, which controlled outreach (in the guise of satellite segregation villages) and case finding.^32^

This gave the institutions for leprosy control an uncommonly high profile as arbiters and interpreters of colonial regulation in southeastern Nigeria; indeed, the restrictions on movement and freedoms from taxation that attached to leprosy inpatient status were frequent points of contestation among patients, missionaries and administrators throughout the 1950s and 1960s.^33^ In 1957, correspondence between the District Office in Uyo and the medical officer of the QIM Leprosy Colony noted the native court sentencing of a supposed patient of the colony clinic for non-payment of taxes. This claim of patient status was referred to the Mission, who clarified that the patient was under treatment but not a regular attendee, noting that ‘it was for his own benefit he was not attending and I do not see why he should be exempt. He came back when it suited him.’^34^ Such cases became increasingly frequent with the transition to outpatient treatment with dapsone from the mid-1950s.

## Missionaries as employers and brokers

The status and propaganda value of these settlements entrenched missionary control of leprosy work, even as it converged in form and function with broader currents in sanitation work, disease control, and preventive and dispensary health. In Ekpene Obom, the story of Bassey Ette, as recounted in the jubilee account of the history of the Qua Iboe Church Leprosy Hospital, exemplifies these trends. Ette grew up and went to school in Ikom, Ogoja Province, and began work with the colonial government before being diagnosed with leprosy and becoming a patient at Itu, the pioneer Church of Scotland settlement on the banks of the Cross River near Uyo. He became head nurse at Itu in the early 1940s, and was transferred to help reopen leprosy work at Ekpene Obom in 1946, where he worked until his retirement in 1974. As with many pioneer African teachers, dressers and nurses in the central settlements, Ette came into paid work through his patient status, and was recognized as an exemplar of love, sacrifice and fidelity. As the authors of the church’s jubilee publication mused, ‘[f]or Bassey and those working with him there was no forty hour week, public holidays, overtime allowances or fringe benefits. Are we any happier today, I wonder?’^35^

The lines of command, between missionary, local government and the Nigeria Leprosy Service continued to be blurred through the late 1950s and 1960s, as leprosy control played its part in opening up and traversing the political, cultural, economic and geographic space of the Kwa Ibo River Basin. O.T Umoh, working in Southern Ukanafun since at least 1953, as a leprosy attendant and later inspector in the employ of the native administration in Ukanafun (from Independence, the Western Annang County Council), was referred to as ‘Our leprosy inspector’ by the QIM medical officer in charge at Ekpene Obom in 1961.^36^ In response to leprosy case discovery resulting from the extension of the Yaws Survey Programme to the Abak area in Northern Annang, Umoh was asked to conduct a leprosy survey there to extend the work he had been doing in Western Annang.^37^

Umoh had previously earned the praise of Kenneth Seal, the leprosy advisor, Nigeria Leprosy Service, Oji River Headquarters, and later rural health advisor, at the conclusion of a long campaign dating back to 1954 to be regraded from native administration leprosy attendant to assistant leprosy inspector. Umoh, a diligent clinic administrator and touring inspector with a good reputation in spite of his lowly grade, had written to Seal in 1958, to invite him to ‘correct and advise’ him to help in ‘building up [his] progress as a trained employee’.^38^ Esther Davis, QIM medical officer in 1960, had intervened on his behalf, noting that the county council may have been blocking his promotion since he was from outside the council area, and that she was attempting to reassign him closer to Eket once she could recruit a suitable replacement.^39^

Subsequent correspondence indicates the political calculations necessary in balancing county politics, missionary advocacy and public health administration. Seal wrote to the Western Annang County Council in November 1960 of the ‘excellent Clinic managed by Mr. Umoh’, recommending his promotion onto a more senior pay scale.^40^ Davis noted in passing in the same letter that a colleague of Umoh’s, Mr Mba – probably O.A. Mbah – had recently begun to show much more enthusiasm for his work with the prospect of being recommended for promotion; though Davis was uncertain whether his merits were as clear as Umoh’s, the need to supervise expansion of clinics might make it worth also promoting Mba. Within this diffuse chain of command, Umoh was able to gain a transfer to Oyubia, in Okobo Oron County, closer to Eket. It appears that aspects of the transfer were seen as irregular, and the Mission medical superintendent needed to smooth the process in writing to both county councils, and secure the intervention of the minister for local government, Eastern Region.^41^

## Isaac Onoh, a Nigerian health bureaucrat as advocate and broker

Isaac Okwara Onoh, who had trained with Kenneth Seal and medical superintendent and research director T.F. Davey at Uzuakoli, was an experienced leprosy control officer, appointed to the Medical Field Unit station of the Ministry of Health branch office at Ikot Ekpene, as leprosy control officer for the Annang counties of Calabar Province.^42^ Onoh’s correspondence with staff at the QIM Leprosy Settlement between 1962 and 1964 consolidates themes emerging in the working lives of the other men working in field leprosy control in western Calabar Province. Onoh acted as a broker for the complaints and representations made by more junior workers in leprosy control, negotiating *per diems* and salaries, promotions and material assistance, touring the counties constantly to review work carried out and prospect for further extension of the apparatus or rural public health, maintaining a system of patient support in the face of bureaucratic reorganization, and ensuring that grants were collected from and receipts issued to county councils across the region.

Missionary medicine often seemed to run according to a providential mode of accounting, where salaries shaded into running costs, a virtue was made of sacrifice and adaptation, and financial reckoning could be deferred or diluted by recourse to a donor public.^43^ But for Onoh and the men he managed, their ability to discharge their responsibilities to work and family, to extend leprosy control and case finding, and simply to move from place to place across an area where colonial rule had been consistently contested and its infrastructure thin on the ground, relied on careful tending of meagre and sporadically replenished financial resources. These economic constraints were foregrounded in the transition to Nigerian management of the apparatus of leprosy control, as issues of cost shaped capacity in an insistent and intricate fashion.

These constraints, and their effects on staff and patients, are manifested especially clearly in discussions of mobility. In his report to Kenneth Seal in October 1962, prior to going out on tour between Uyo and Oron, Isaac Onoh noted that the Arochuku [sic] division and outlying districts around Uyo were difficult to reach and supervise, and that the areas around Calabar were not yet properly covered or supervised.^44^ The reorganization of rural public health in the 1960s meant that leprosy attendants were less likely to be drawn from local or patient communities, which had implications for mobility both while working and going on leave. Leprosy inspectors were often expected to cover both yaws and leprosy survey work, which imposed onerous and expensive transport and accommodation schedules on them. Advances were available but often very slow in being paid, such that Onoh was required to write frequently to the Rural Health Headquarters in Oji River as well as the QIM Leprosy Settlement in Ekpene Obom and various county councils in Western Calabar Province to maintain mobility and inspection schedules.

E.E. Ecoma, acting rural health adviser at Oji River, wrote to Onoh in May 1962 that a leprosy inspector needed to be released to cover Uyo, and should apply for a motorcycle to work on the same basis as the inspector for Abak, who had a motorcycle to enable ‘the integration of the yaws and leprosy services for purposes of efficiency and economy’. Onoh’s reply pointed out that S.I. Adim, the acting district leprosy inspector at Abak, had not received his motorcycle advance, and would need nine nights’ sleeping out allowance to cover the cost of his upcoming yaws resurvey work.^45^ When Onoh himself went on tour, he encountered many of the same problems experienced routinely by his cadre of inspectors. Opobo and Obolo, either side of the mouth of the Imo River to the west of the Kwa Ibo Basin, were part of the expansion programme in leprosy control overseen by Onoh, and could only be reached by renting the government launch (a small open motorboat), which Onoh had to book with two months’ notice.^46^ On his return from this tour, he felt obliged to request an increased mileage allowance, from 700 to 1000 miles per month, to fulfill routine duties and to attend county council meetings on request to lecture on leprosy control, pleading that ‘[w]here such invitations are not followed up, Councils often have enough grounds to refuse leprosy work in their areas.’^47^ He was to eventually recommend that new appointees possess motorcycles, due to difficulties in approving motorcycle advances.^48^

The principle of support for worker mobility needed assiduous advocacy. Here, the Qua Iboe Mission also played a key role as a PAYE employer (income taxed at source), pointing out the inequities involved in a revenue policy of taxing transport allowances unless specifically exempted. In June 1962, the Regional Ministry of Finance confirmed on request that tax need not be deducted from employee transport allowances. Both the Mission and the leprosy inspectors sought relief for patients, either through clarifying exemptions from bicycle tax (collected at district council level), or by arranging the provision of leprosy services in the existing dispensary system so that patients need not travel far for treatment. Travelling to Obolo to begin outpatient treatment for five patients at Ngo dispensary, A.K. Udochu, the divisional leprosy inspector for Opobo expected and requested free transport on the council’s launch as *quid pro quo* for obliging the patients.^49^

The diffuse lines of responsibility for financing and administering leprosy control impinged on the everyday and family lives of leprosy inspectors and attendants. The rental of a QIM Land Rover to allow O.T. Umoh to go on leave in January 1961 had still not been covered by Western Annang District Council 13 months later.^50^ For A.K. Udochu, there was no clear line of recompense for out-of-pocket expenses for essential office equipment including official stamps and a survey register. Onoh annotated and forwarded Udochu’s claim to Ekpene Obom, claiming ‘Govt. has no funds for this and I refer it to you in the hope that you may help please.’^51^ This annotation was delivered with a letter asking for about six gallons of petrol monthly to be given to Udochu to augment a 250-mile government allowance and enable him to cover Eket alongside extensive yaws duties in Ibibio, Four Groups and Annang County Council areas. Onoh noted that Udochu was covering between 500 and 700 miles monthly, ‘and I know he is suffering in silence (financially)’.^52^

## Disease control politics and the precarity of labour

When seeking to explain the uneven and contested spread of leprosy control in Calabar Province, where some areas with endemic leprosy were only beginning to be covered in the 1960s, we also need to take into account the deep distrust of inspection, case detection and other forensic labours associated with the state in the late colonial era, when violent disorder, active disruption of local economic relations, and recourse to Annang and Ibibio forms of restitution and retribution characterized much of the local political terrain. This distrust was dramatized by the fallout from the posting of Fred Akpan to Northern Annang County Council in Abak. Akpan was a Mission employee, who had been trained at the Rural Health Training School (formerly the Leprosy Inspectors Training School) at Oji River and at Ekpene Obom before being posted to Abak as a leprosy attendant. As well as regularly underestimating the cost of leprosy control and patient support at the central settlement in Ekpene Obom, Northern Annang County Council dragged its feet on taking over the contract for Akpan from the Mission.

The disputed status of leprosy work in the area was underscored when Akpan attended the leprosy clinic within the former town hall at Ito Ika. Local residents seem to have objected to the treatment of leprosy in the dispensary; Akpan was

beaten up … a whole tin of Dapsone [leprosy treatment] tablets … were alleged seized. The treatment register was torn to pieces while Mr Akpan sustained injuries for which the Abak police has led him to Ikot Ekpene General Hospital for treatment.^53^

When the case was heard, the Ito community cited the 1916 Leprosy Ordinance mandating segregation of leprosy patients as a defence of its violent attack. Though the Ordinance had been repealed in 1957, under pressure from leprosy workers and the WHO, the defence was accepted by the court and charges were dropped against the community.^54^ Onoh advised Akpan, whose contract still hadn’t been regularized by the council:

You are in no way deserted in this matter … . At the moment, I will suggest that you keep treating the patients at the present place until the District Officer, Abak, Dr. Seal and I, shall have discussed the matter further with the Ito people … . May be [sic] peace will return at least between us and the village … I doubt if it is possible to transfer you to another place. We have asked the County council to appoint you … . If you are a little tolerant, patient, diplomatic and friendly. Depending on our advice and sound judgment all the time, it is possible that when sentiment dies down, the people will forget the matter and cooperate with us.^55^

For Fred Akpan, contractual uncertainty was compounded by being required to conduct his work amid physical insecurity, as the means to extend leprosy control was undermined by funding difficulties and violent local resistance. His case was a stark reminder of the unevenness of rural public health extension work, of the availability of funds and resources, and of the micropolitics of government intervention on a terrain where state agency was rarely experienced as even-handed. Caught between missionary and government hierarchies, Akpan’s case was an eloquent expression of the precarious nature of employment in leprosy control as lines of responsibility transferred and tangled between county council, Ministry of Health and medical missionary.

## Employing health care workers and paying for leprosy control

Many of the difficulties encountered in their everyday working lives by the men supported and directed by Isaac Onoh arose from the complexities of devising and funding a structure for outpatient leprosy control. Missionaries had become expert in developing settlement-based outreach on a relative shoestring, and the rhetorical value of the central settlement, as a showcase of Christian charity, enabled missionaries to make repeated pleas for international donor support, as well as to build a case for government support through the management of a vital health service. However, the principle of a leprosy control service run distinct from and in parallel with rural public health had come to violate new integrative modes in administering rural public health.

Rather than explicitly supplanting missionary-run leprosy control programmes, an initially parallel bureaucracy of rural public health instituted new command structures based on a broader programme of administrative reorganization in late colonial Nigeria. Missionary resources, investments and expertise were not to be sidelined, but rather harnessed to the project of integrating leprosy control with medical, social and welfare provision across Eastern Nigeria. As leprosy control officer within a larger Medical Field Unit, Onoh was able to rely on and develop a holistic vision of this process of integration, using it to support leprosy work wherever possible. He was especially keen to enlist the assistance of divisional social workers in resettling discharged leprosy patients, using their prestige to help overcome social issues facing leprosy patients outside the settlement system.^56^

Indeed, the work of ensuring that county councils committed funds promised to leprosy control in their estimates rested largely on the moral authority of the medical superintendent of the missionary leprosy settlement. Writing to the divisional officer in Abak to follow up on a consistent theme in correspondence with Onoh, and on a meeting in person earlier in the month, the missionary superintendent presented a series of ‘facts’ about the scale of leprosy control and the funding deficits in the various Annang counties. It was clear that some of the leprosy inspectorate in the counties was still employed by missionaries, who also had responsibility for maintaining ‘pauper patients’. The QIM made claims on the councils for patient maintenance, staff employment and materials used in rehabilitation and care of patients, noting that some of the councils had failed to make provision for leprosy expenditure, and others had not provided funds promised.^57^ The QIM in turn hoped to engage the agency and authority of the leprosy control officer to secure early payment of grants.^58^

This echoed the experience of Onoh, whose tabulation of leprosy grants for the missionary medical superintendents showed a disjuncture between the scale of needs and the commitment of funds in various councils, some of which could be accounted for by poverty, and some by the relatively low priority accorded to leprosy by autonomous county councils.^59^ The disjuncture was reflected in his report of September 1962 to the rural health advisor, Oji River, where the delicate balancing act between coverage, infrastructure, funding and labour capacity was set out in some detail. He noted the work of S.I. Adim, and praised the friendly approach of A.O. Ubbor, while pleading that the workload needed greater human resources for both supervision and extension.^60^ In the case of A.K. Udochu, he made an explicit complaint to the Ministry of Health that Udochu was expected to cover a far greater territorial expanse than ought to be allotted to a single district leprosy inspector.^61^

By 1964, after two years in post, Onoh had managed to engage directly with all the county councils in the field area, involving them more fully in leprosy control as a component of administration and governance. Reminding councils and councillors of their responsibility for the support of patients and the funding of clinics, Onoh managed to streamline the administration of leprosy control as an element of broader medical field work and rural public health. What had been a network of relatively *ad hoc* leprosy clinics emanating from missionary-run central settlements, expanding as and when resources permitted, now took on the appearance of a service which was part and parcel of government commitments to rural populations regardless of distance from the nuclear settlement.

In his final handing-over note on leaving his post in October 1964, Onoh described the administrative terrain that would greet his successor. In doing so, he illustrated the extent to which the Medical Field Unit had come to assume authority within the administration of leprosy control. Referring to the medical superintendents of the missionary leprosy settlements at Itu and Ekpene Obom, he noted that it ‘is necessary to consult [them] from time to time especially re the time for examining of patients’. He spoke of the officer’s responsibility for assigning UNICEF motorcycles, for visiting dispensaries to attend notified cases of leprosy, and for collecting grants from county councils, with the assistance of missionary medical superintendents, whom it was presumed could exert moral pressure, since hospitalized cases had always been the responsibility of native administrations and their successor county councils. Transport was still the major funding and allowances headache, and the continuing work of the Yaws Initial Treatment Survey in Uyo Division was bound to be of keen interest to the incoming officer.^62^

## Conclusion

Contemporary accounts of missionary evangelical and medical enterprise in colonial Africa focused on tropes of increase and redemption to explain patterns of institutional development. Thus, in missionary accounts, leprosy control took root and spread organically from its halting beginnings, through the force of its own innate appeal with little reference to local histories, obstacles and determinants. David Pratten shows how this account is likely to founder in and around Ukanafun and Ibesit in Western Annang, given the turbulent political economy of colonial conquest and pacification in the region. Over the period from the early to mid-1950s through to the late 1960s, missionary engagement with local administrations changed substantially, both in tone and in the expectations vested in the relation. These changes registered in the management of finances as well as of staffing and chains of responsibility. Both missionary and local administrators continued to collate and voice complaints, but the locus of stability underpinning the delivery and expansion of leprosy control was less in the mission itself and more in the cadres of inspectors and the apparatus for the design and delivery of rural public health.

This was a terrain in which leprosy workers had to proceed carefully and cautiously, always attentive to questions of reputation, probity and responsibility which could easily disrupt plans, calendars and the financial lifeline on which the work of leprosy control relied. Echoing experiences across colonial Africa, and identifying common issues of labour in public health at the end of empire, the precarity of work in leprosy control and rural public health emerges starkly from the strands of correspondence analysed here. Anxieties about vocations and careers in leprosy control, in uneven transition from colonial to Nigerian stewardship, remained unresolved throughout the period, while personal security and issues of performance and supervision, compensation, status and protection continued to loom large in the lives and work of the men entrusted with the work of extending leprosy control and rural public health work throughout the region.

The bureaucratic hierarchy of leprosy control blended lines of responsibility to the medical superintendents of historically missionary leprosy settlements (Itu and Ekpene Obom in the case of Calabar Province) and the regional Ministry of Health of the government of Eastern Nigeria. The lines converged in the person of the leprosy control officer, who linked district leprosy inspectors to the leprosy settlements, their status, salary and promotions to the ministry, and their call on resources fringed across both lines of responsibility. In an expanding terrain of leprosy control, local government expectations and finances also needed careful and repeated attention, to maintain the viability of the enterprise at its most intimate and local levels.

Convincing local councils of the value of leprosy control and the need to meet its cost was an ongoing battle, demanding all the wiles and vigour of the inspectorate, together with a consistent propaganda effort as well as advocacy on behalf of the patients from a locality. As patients might not be receiving adequate care at home, or be left destitute in central settlements without budgetary commitments by councils, considerable political effort was put into making leprosy and its control a routine component of annual budgeting in the areas covered by provincial leprosy control officers and district leprosy inspectors; as their reach increased in the 1960s the viability of leprosy control apparatus was always under great strain, either through over-commitment or impaired funding flows. Both strains played out prominently in the working lives of the inspectorate.

In colonial public health, the inspectorate in disease control is seen as a group to be monitored, policed and directed. Sleeping sickness and early mobile survey units were used as a resource to be redeployed within yaws eradication work in the early 1950s, rather than a group whose agency might play a shaping role in negotiating and producing public health outcomes. This is an environment in which cost *control* is a key component of the policy terrain. But the control of disease was shaped and directed by their intimate responses and relations to communities and people presenting with ill health in all of its contrary social forms. In this, the experience of public health workers in leprosy control in the Kwa Ibo River Basin exemplifies processes described by Lachenal et al., Hunt, and Kalusa, where medical workers act as interpreters, translators, brokers and neighbours in late colonial and independent Africa.^63^

Anxieties over the spread of leprosy, the cost of leprosy and the social toll of leprosy recurred throughout the experiences of leprosy workers, bureaucrats, missionaries, patients and their communities of origin. Here, the anxieties are manifested in correspondence about the management of outpatient leprosy control and referral between the QIM Leprosy Hospital in Ekpene Obom, the county councils, and senior leprosy workers in Annang and Ibibio areas of Calabar Province. These struggles and anxieties, as well as the adaptations and resilience of fieldworkers in leprosy control are central to our understanding of how spatial, territorial and trans-territorial contexts of leprosy control and rural public health were operationalized on the ground in and around the Kwa Ibo River Basin, and Eastern Nigeria more broadly, in the 1960s.

## Notes

1. In the archival correspondence examined for this article, the terms province, district, division and county council are often interchanged according to context. In colonial and early independent Nigeria through to 1967, the highest territorial unit had been the province, until this was superseded in 1953 by the division of the colony into three federal regions. Calabar Province, which continued to exist until 1967, was part of the Eastern Region headquartered in Enugu. Normally, provinces were divided into colonially administered (i.e. by British officers) districts and divisions. In the Eastern Region, local political expression was organized via clan courts and native administrations. By 1960, the native administrations had been reorganized into county councils. Some confusion arises in the archive when field divisions for leprosy control are referred to as provinces (e.g. Uyo Province, Abak Province). Since these designations do not correspond to mainstream administrative territories, I have not used province in the text where it refers to a leprosy control province. Correspondents routinely navigated the overlapping designations as a part of the terrain in which they worked, and the colonial designations of district and division were only slowly phased out from job titles and popular usage in the early Independence period.

2. Gbadamosi and Davis, *A Symbol of Love and Sacrifice*, 1–7.

3. Pratten, *The Man-Leopard Murders*, 74–81, 285–6; and Abasiattai, *A History of the Cross River Region of Nigeria*, chapter 13.

4. Northrup, *Trade without Rulers*, 224–30.

5. Afigbo, “A Reassessment of the Historiography of the Colonial Period in Anglophone West Africa,” 409–18; and Forrest, *The Advance of African Capital*, 13–22.

6. Gbadamosi and Davis, *A Symbol of Love and Sacrifice*, 5. Nigeria National Archives, Enugu (NNAE). CALPROF 3/1/745 Qua Iboe Mission leper [sic] colony at Ekpene Obom, Uyo Dist. Letter from J.M. Westgarth, Qua Iboe Mission to Divisional Officer, Uyo, dated December 26, 1933.

7. NNAE CALPROF 3/1/745 Qua Iboe Mission leper [sic] colony at Ekpene Obom, Uyo Dist. Report on leprosy work at Ekpene Obom, sent by W.H. Graddon, Secretary, Qua Iboe Mission, Eket, to C. Mayne, Resident, Calabar Province, dated July 5, 1950. Letter, ‘Grant-in-aid: leprosy control’, from R.H. Bland, Senior Leprosy Officer, Nigeria Leprosy Service Headquarters, Oji River, to the Resident, Calabar Province, dated February 12, 1951.

8. Heap, “The Nigerian National Archives, Enugu,” 434; and Akoda, “Using the Archives for Research,” 76–8.

9. Litsios, “The Christian Medical Commission and the Development of the World Health Organization’s Primary Health Care Approach,” 1887–8; and Cueto, “The ORIGINS of Primary Health Care and SELECTIVE Primary Health Care,” 1865.

10. Gorsky and Sirrs, “From ‘Planning’ to ‘Systems Analysis,’” 206–10; and Manton and Gorsky, “Health Planning in 1960s Africa.”

11. Bhattacharya and Campani, “Re-Assessing the Foundations,” 88–93.

12. See Geissler et al., “Introduction” for a useful discussion of the role of auxiliary health and sanitation workers in nurturing civic and political life in postcolonial Africa, which picks up on similar themes to those raised here.

13. Ityavyar, “Background to the Development of Health Services in Nigeria,” 493–6.

14. World Health Organization Headquarters (WHO HQ) Archives, Geneva. T2/372/10 Programming difficulties concerning UK territory projects. Memorandum ‘Problems connected with projects planned or in operation in U.K. territories’, from the Regional Director, WPRO to the Director General, Geneva, dated September 14, 1956, outlines continued bureaucratic uncertainty and obstruction from the UK Colonial Office on accepting technical assistance and programming from WHO.

15. Zahra, “Yaws Eradication Campaign in Nsukka Division, Eastern Nigeria,” 913, 930.

16. Nigeria National Archives, Ibadan (NNAI) MH(FED)1-7-D/14150-S.1 WHO sixth regional committee projects. Letter headed ‘1956 W.H.O. Regional Meeting’ from the Director of Medical Services, Eastern Region, Enugu to the Chief Medical Advisor to the Federal Government, Lagos, dated August 20, 1956.

17. Ibid., 913–18; Manton, “Leprosy in Nigeria and the Social History of Colonial Skin,” 130–1.

18. Kalusa, “Language, Medical Auxiliaries, and the Re-Interpretation of Missionary Medicine in Colonial Mwinilunga, Zambia, 1922–511,” 59–60; Kalusa, “From an Agency of Cultural Destruction to an Agency of Public Health,” 236–8; Hunt, *A Colonial Lexicon of Birth Ritual, Medicalization, and Mobility in the Congo*; Lachenal et al., “Neglected Actors in Neglected Tropical Diseases Research”; and Greenwood, *Beyond the State*.

19. Manton, “Leprosy in Nigeria and the Social History of Colonial Skin,” 132; Manton, *The Roman Catholic Mission and Leprosy Control in Colonial Ogoja*, chapter 3; Shankar, “Medical Missionaries and Modernizing Emirs in Colonial Hausaland,” 51–3; and Vongsathorn, “First and Foremost the Evangelist?,” 551–4.

20. Manton, “Leprosy in Nigeria and the Social History of Colonial Skin”; Gradmann and Mbuya, “Kibong’oto”; Davey, “A Study of Leprosy in South Eastern Nigeria (with Special Reference to the Owerri Province)”; and Davey, Ross, and Nicholson, “Leprosy.”

21. Pratten, *The Man-Leopard Murders*, 82–99.

22. Cueto, “The ORIGINS of Primary Health Care and SELECTIVE Primary Health Care.”

23. Manton, “The Roman Catholic Mission and Leprosy Control in Colonial Ogoja,” chapter 3.

24. Kalu, *Clio in a Sacred Garb*, 207–8.

25. Muir, “Leprosy in Nigeria.”

26. Falola, *Development Planning and Decolonization in Nigeria*, 108.

27. Drafting Committee. “Conference on Control of Yaws, ‘Report of the Second International Conference on Control of Yaws: Nigeria, 1955 – Part I’”; and “Conference on Control of Yaws, ‘Report of the Second International Conference on Control of Yaws: Nigeria, 1955 – Part II.’”

28. Zahra, “Yaws Eradication Campaign in Nsukka Division, Eastern Nigeria,” 931.

29. Zahra, “Yaws Eradication Campaign in Nsukka Division, Eastern Nigeria,” 930.

30. Ogoja Convent files. Minutes of the [fifth?] meeting of the Eastern Region Leprosy Advisory Committee [sic], February [1956], paragraph 6.

31. Manton, “The Roman Catholic Mission and Leprosy Control in Colonial Ogoja,” 97, n. 51; and Muir, “Leprosy in Nigeria,” 155, 166.

32. Manton, “The Roman Catholic Mission and Leprosy Control in Colonial Ogoja,” chapter 5.

33. Manton, “The Lost Province,” 344–5.

34. Ekpene Obom Files. Letter from J. Richardson, Administrative Officer, District Office, Uyo to the Medical Officer, QIM Leper Clinic [sic], dated April 9, 1957 and replies dated April 17 and April 20, 1957.

35. Gbadamosi and Davis, *A Symbol of Love and Sacrifice*, 22–3.

36. Ekpene Obom files. Letter from the Medical Officer in Charge, QIM Leprosy Settlement to the Secretary, Northern Annang County Council, Abak, dated November 20, 1961.

37. Ibid. and Ekpene Obom files. Letter from K. Seal, Rural Health Division, Oji River, to the Area Superintendent, Rural Health Division, Uzuakoli, dated October 26, 1961.

38. Ekpene Obom files. Letter from O.T. Umoh, Ikot Otor Iwo Leprosy Clinic to the Leprosy Adviser, Oji River, dated November 28, 1958.

39. Ekpene Obom files. Letter from Esther Davis, Medical Officer, QIM Leprosy Settlement to K.S. Seal, Oji River, dated October 24, 1960.

40. Ekpene Obom files. Letter from K.S. Seal, Rural Health Division, Oji River to the Secretary, County Council Offices, Ukanafun, Western Annang, dated November 10, 1960.

41. Ekpene Obom files. Letters from the Medical Officer, QIM Leprosy Settlement to the Minister of Local Government, Enugu dated March 21, 1962 and March 26, 1962; from the Medical Superintendent, QIM Leprosy Settlement to the Secretary, Western Annang County Council dated April 10, 1962; from the Permanent Secretary, Ministry of Local Government, Enugu to the Medical Officer, QIM Leprosy Settlement dated April 13, 1962; and from the Secretary, Western Annang County Council to the Medical Superintendent, QIM Leprosy Settlement dated April 17, 1962.

42. Interview with Isaac Okwara Onoh, March 18, 2015.

43. Manton, “The Roman Catholic Mission and Leprosy Control in Colonial Ogoja,” chapters 5 and 6; Macdonald, *Can Ghosts Arise?*; Chukwu and Ekekezie, *The Leprosy Centre Uzuakoli (1932–1992)*; Gbadamosi and Davis, *A Symbol of Love and Sacrifice*; and Medical Missionaries of Mary, *The First Decade*. [These works all contribute to an impression of the imagined role of providence and sacrifice in sustaining leprosy control work].

44. Ekpene Obom files. Letter, ‘Leprosy Control Unit – S.E. Zone Report for September, 1962’ from Isaac Onoh, Leprosy Control Officer, Ikot Ekpene to Kenneth Seal, Rural Health Advisor, Oji River dated October 10, 1962.

45. Ekpene Obom files. Letter from Isaac Onoh to the Medical Superintendents, Itu and Ekpene Obom, dated June 5, 1962, quoting letter from E. E. Ecoma dated May 25, 1962. Letter from Isaac Onoh to E.E. Ecoma, Rural Health Headquarters, Oji River, dated June 5, 1962.

46. Ekpene Obom files. Letter, ‘Government Launch. Application for,’ from Isaac Onoh to the Divisional Officer, Opobo, dated July 6, 1962 requested the use of the launch for Onoh and the District Leprosy Inspector for Opobo on September 6 and 8, 1962.

47. Ekpene Obom files. Letter from Isaac Onoh to the Rural Health Advisor, Oji River, dated September 18, 1962.

48. Ekpene Obom files. Letter from Isaac Onoh to the Rural Health Adviser, Oji River, dated March 12, 1963.

49. Ekpene Obom files. Letter from the Secretary QIM Leprosy Settlement to the Commissioner of Internal Revenue, Ministry of Finance, Enugu, dated November 1, 1962 and reply dated November 12, 1962, and letter from A.K. Udochu to The Secretary/Treasurer, Obolo County Council, dated March 25, 1963.

50. Ekpene Obom files. Letter from the Sister-in-Charge, QIM Leprosy Settlement, to the Secretary Treasurer, Western Annang County Council, S. Ukanafun, dated January 25, 1961 and from the Secretary, QIM Leprosy Settlement to the Secretary, Western Annang County Council, dated February 28, 1962.

51. Ekpene Obom files. Letter from A.K. Udochu, District Leprosy Inspector, Opobo to Isaac Onoh, dated April 22, 1963, with an annotation to the Sister-in-Charge, QIM Leprosy Settlement, Ekpene Obom, dated April 24, 1963.

52. Ekpene Obom files. Letter from Isaac Onoh to the Medical Superintendent, QIM Leprosy Settlement, dated April 24, 1963.

53. Ekpene Obom files. Letter from the Secretary, Northern Annang County Council to the Leprosy Inspector, Utu Etim Ikpe dated April 12, 1962, granting permission to use the former Ito Native Court Hall for a leprosy clinic. Letter from Isaac Onoh to the Medical Superintendent, QIM Leprosy Settlement, dated October 25, 1962.

54. See n. 48.

55. Ekpene Obom files. Letter from Isaac Onoh to Fred Akpan, dated March 2, 1963.

56. Ekpene Obom files. Letter from Isaac Onoh to the Rural Health Adviser, Oji River, dated July 6, 1962.

57. Ekpene Obom files. Letter from the Medical Superintendent, QIM Leprosy Settlement to the Divisional Officer, Abak dated July 27, 1962.

58. Ekpene Obom files. Letter from Esther Davis, Medical Superintendent, QIM Leprosy Settlement to the Secretary, Northern Annang County Council, dated April 12, 1962.

59. Ekpene Obom files. Letter from Isaac Onoh to the Medical Superintendents, Itu and Ekpene Obom, dated November 12, 1962.

60. See n. 44.

61. Ekpene Obom files. Letter from Isaac Onoh to the Area Superintendent, Ministry of Health, Uzuakoli dated June 10, 1962.

62. Ekpene Obom files. Handing Over note from Isaac Onoh to D.E. Ukauwa, dated October 8, 1964.

63. Kalusa, “From an Agency of Cultural Destruction to an Agency of Public Health”; Hunt, *A Colonial Lexicon of Birth Ritual, Medicalization, and Mobility in the Congo*; Lachenal et al., “Neglected Actors in Neglected Tropical Diseases Research”; and Greenwood, *Beyond the State*.
